# Random forest approach to predict genes correlated with adaptive responses to KRAS^G12C^ inhibitor

**DOI:** 10.1371/journal.pone.0359938

**Published:** 2026-11-11

**Authors:** Gregory P. Riddick, Allison Pittman, Stephanie S. McHale, Parash Parajuli, Bin Zhu, Michelle Van Scoyk, Pei-Ying Wu, Chu-Fang Chou, Hyeong-Min Lee, Robert A. Winn

**Affiliations:** 1 Department of Internal Medicine, School of Medicine, Virginia Commonwealth University, Richmond, Virginia, United States of America; 2 Medical Scientist Training Program, School of Medicine, Virginia Commonwealth University, Richmond, Virginia, United States of America; 3 Massey Comprehensive Cancer Center, Virginia Commonwealth University, Richmond, Virginia, United States of America; 4 Department of Cell, Molecular, and Genetic Medicine, School of Medicine, Virginia Commonwealth University, Richmond, Virginia, United States of America; 5 Research Institute of Fox Chase Cancer Center, Philadelphia, Pennsylvania, United States of America; 6 Department of Cancer and Cellular Biology, Lewis Katz School of Medicine, Temple University, Philadelphia, Pennsylvania, United States of America; University of Michigan, UNITED STATES OF AMERICA

## Abstract

KRAS mutations represent one of the most common and clinically challenging oncogenic drivers in non–small cell lung cancer (NSCLC), a leading cause of cancer mortality. The recent development of KRAS inhibitors, particularly targeting KRAS^G12C^ has transformed the therapeutic landscape. However, despite these advances, treatment benefits remain limited as most patients experience disease progression within months due to adaptive or acquired resistance mechanisms. The incomplete understanding of the adaptive and acquired resistance mechanisms restricts our progression toward the development of effective therapeutic intervention. Particularly, we questioned whether common, targetable molecular vulnerabilities exist across distinct KRAS^G12C^ tumor contexts during the early adaptive window under therapeutic pressure, preceding the establishment of overt drug resistance. To address this, we employed a Random Forest to single-cell RNA sequencing data from three KRAS^G12C^ mutant NSCLC cell lines, enabling systematic ranking of genes associated with proliferative versus quiescent cell-state annotations. Our analysis recapitulated established proliferation-associated genes at baseline and revealed distinct gene signatures emerging at 72 hours of treatment, enriched for translational control and other biological pathways. Notably, *PHF19* and *TMEM106C* were consistently prioritized across cell lines and showed higher direct expression in the proliferating populations, supporting their designation as candidate correlates of the residual proliferative state during early adaptation. These findings define proliferation and differentiation as a potential early vulnerability in KRAS^G12C^ mutant NSCLC and provide a conceptual framework for intercepting resistance development, requiring subsequent external and functional validation.

## Introduction

Despite advances in targeted therapies and immunotherapies, non-small cell lung cancer (NSCLC) remains one of the deadliest malignancies. It accounts for approximately 85% of all lung cancer cases [1,2]. Particularly in patients with specific genetic mutations, such as KRAS (Kirsten Rat Sarcoma) ^G12C^, or ^G12D^, NSCLC has been associated with poor clinical outcomes [3–7]. The five-year survival rate for NSCLC hovers around 25–32% [8], representing the urgent need for more effective therapeutic strategies and a fundamental understanding of the molecular mechanisms driving tumor initiation, progression, and treatment resistance.

KRAS, which encodes a signaling protein in the RAS-MAPK signaling pathway, plays a key role in the initiation of NSCLC and is the most commonly mutated oncogene, found in approximately 30% of NSCLC patients [9–12]. KRAS mutations result in constitutive activation of downstream signaling cascades that promote uncontrolled cell proliferation, survival, and metastasis [9–12]. For decades, KRAS-mutant cancers have been associated with particularly poor prognoses and limited therapeutic options [4,11]. Due to its structural surface and high affinity for GTP, KRAS was recognized as “hard to be druggable” [13,14], which posed a significant challenge for traditional small-molecule inhibition. This paradigm shifted dramatically with the development of covalent inhibitors selectively targeting the KRAS^G12C^ mutation, the most common KRAS variant in NSCLC. Drugs such as AMG510 (sotorasib) and MRTX849 (adagrasib) bind irreversibly to the mutant cysteine residue, effectively locking the KRAS^G12C^ protein in its inactive GDP-bound state [15–18]. Furthermore, MRTX1133 is the first noncovalent and selective inhibitor for KRAS^G12D^ [12,19]. Pan/multi-RAS/KRAS inhibitors have also been proven to prevent the activation of a broad range of KRAS mutants in the preclinical phase [12,20]. The FDA approval of sotorasib in 2021 [21], followed by the approval of adagrasib [22] represented breakthrough therapies, finally bringing hope to patients with KRAS^G12C^ mutant NSCLC. These inhibitors demonstrated remarkable initial clinical responses, with objective response rates ranging from 30–40% in heavily pretreated patients [17,23–25].

However, drug resistance has limited the initial success of these targeted therapies. Most patients undergo disease progression within 6–12 months of treatment initiation, as tumors evolve by employing diverse mechanisms to bypass KRAS^G12C^ inhibition [23,26–28]. This pattern is frequently observed in other targeted therapies in oncology, where initial responses are followed by adaptive resistance, and eventual therapeutic ineffectiveness. The mechanisms underlying this resistance are complex and multifaceted, involving both on-target alterations (such as secondary KRAS mutations), and off-target bypass pathways that reactivate proliferative signaling [23,26–28]. Previous reports suggest that on-target resistance through acquired KRAS mutations predominates in certain patient populations [26,29], while other studies emphasize the importance of bypass pathway activation through receptor tyrosine kinase upregulation or downstream effector reactivation [30–32]. This broader challenge in precision oncology can be remedied by understanding and addressing whether resistance mechanisms are patient-specific, requiring personalized combination strategies, or whether common vulnerabilities exist that can be exploited therapeutically across diverse genetic backgrounds. Furthermore, understanding these resistance mechanisms is critical for developing combination strategies, sequential therapies, and next-generation inhibitors that can provide more durable clinical benefit.

In this study, by employing a computational approach to delineate transcriptomic landscape, we identified both reported and emerging genes involved in early adaptation under therapeutic pressure. Our analysis revealed *PHF19* (PHD Finger Protein 19) and *TMEM106C* (Transmembrane Protein 106C) as emerging early adaptation genes, and cell cycle progression and proliferation drivers, for examples, *H2AFZ, CKS1B, TUBA1B, RRM2,* and *BIRC5* as well-documented genes. Targeting the emerging markers potentially provides a window of opportunity to intercept resistance before it matures. However, feature importance measures association rather than causality, these candidates are presented as hypotheses for subsequent functional testing rather than established early-adaptation drivers or therapeutic targets. Thus, future experimental validation of these genes will offer a proactive strategy to improve clinical outcomes rather than attempting to overcome KRAS^G12C^ resistance in NSCLC patients.

## Materials and methods

### Rationale of computational modeling

To identify critical genes under therapeutic pressure in KRAS^G12C^-mutant lung cancer, our methodology was designed to capture the dynamic transcriptional landscape of cancer cells, with particular attention to early adaptation changes occurring under therapeutic pressure. We utilized scRNA sequencing data (GSE137912) derived from three independent KRAS^G12C^-mutant lung cancer cell lines: H358, H2122, and SW1573 treated with 10 μM of ARS-1620 [33]. These cell lines were selected based on their validated KRAS^G12C^ mutation status and their widespread use in preclinical studies of KRAS inhibitors [33,34]. To capture early adaptation changes induced by ARS-1620, we selected the datasets at two time points, 0 hr (T0) and 72 hr (T72) post treatment, representing “untreated” and “early adaptive development”, respectively. This temporal design allowed us to capture both constitutive proliferation and emergent adaptive programs.

### Model selection and training

We implemented a Random Forest machine learning algorithm to classify cells as either proliferative or quiescent based on their gene expression profiles, using the ranger engine in the parsnip package of the tidymodels framework in R (version 4.5.2, 2025-10-31) using RStudio (version 2025.09.2 + 418). Briefly, we used the cell-level annotations accompanying GSE137912, as generated by Xue et al [33]. In the deposited annotation, G0 cells are designated quiescent, whereas cells assigned to the G1S, S, G2M, M, or MG1 phases are designated proliferating. We restricted this annotated dataset to the H358, H2122, and SW1573 cells collected at T0 and T72, the two treatment time points examined in our study. Random Forest was chosen for its robust performance in high-dimensional biological data, its ability to capture non-linear relationships between genes, and its inherent feature importance ranking capability. The model was trained using labeled examples of proliferative and quiescent cells, learning to distinguish the transcriptional signatures associated with each state. Each cell line–time-point dataset was partitioned independently into training, validation, and test subsets at an approximately 70:15:15 ratio. Stratified random sampling based on the published binary cell-state label (Mode) was performed using a fixed seed of 12345, thereby preserving the proliferating-to-quiescent distribution across subsets. Cell line and time point were controlled by fitting separate models for each condition and were therefore not used as stratification variables. Sequencing depth was not used during partitioning, and cell identifiers were mutually exclusive across subsets.

### Gene ranking methodology

To rank genes based on their contribution to accurate classification of proliferative versus quiescent states, we leveraged the Random Forest’s built-in feature importance metrics after model training using the random forest function with the ranger engine and ranking importance via impurities which was performed in the tidymodels framework in RStudio. For each cell line and time point, the models were fitted using all gene-expression features (8,687 genes) retained in the processed GSE137912 scRNA-seq expression, allowing us to identify both common proliferation genes and context-specific genes that may represent adaptive development mechanisms. Each forest contained 1,000 trees, with 93 variables randomly considered at each split (mtry = 93, corresponding to the integer square root of 8,687), a minimum node size of 1, the Gini impurity split rule, unrestricted tree depth, and bootstrap sampling with replacement at a sampling fraction of 1.0. Random Forest construction did not use an iterative numerical convergence threshold. Training terminated after establishing the prespecified 1,000 trees. After the model settings were fixed, the evaluation forest was fitted using the combined training and validation subsets, and the test subset was used once for final held-out evaluation. Following model fitting, genes were ranked in descending order according to impurity-based feature importance, which represents the cumulative contribution of each gene to reducing classification impurity across the decision trees. The top 1,000 importance-ranked genes from each model were retained as an exploratory set for downstream overlap, functional-enrichment, and network analyses. Since genes with higher importance metrics served as being essential for decreasing the impurities in the classification of a proliferative phenotype, we categorized four tiers based on the score: top (90–100%, main contributor), upper-middle (65–90%, strong contextual contributor), lower-middle (30–65%, backup), and bottom (0–30%, least contributor). Those ranked genes at each time point were further examined for biological and functional associations using STRING (string-db.org) [35]. Protein interaction networks were partitioned using the STRING K-means clustering option with a predefined number of clusters (k = 3–6). In STRING, this procedure is implemented using a k-medoids algorithm applied to a network-derived distance matrix, such that proteins with stronger or closer functional associations are preferentially assigned to the same cluster. The resulting clusters were used to identify functionally coherent network modules and to improve visualization of the larger interaction networks.

## Results

### Model performance assessment

We followed our workflow to identify critical genes under therapeutic pressure in KRAS^G12C^-mutant lung cancer (Fig 1A). We evaluated the performance of our Random Forest classification model in distinguishing proliferative from quiescent cells across all cell lines and time points. At T0, proliferating cells predominated in H358 (974/1,079), H2122 (412/482), and SW1573 (916/1,004). At T72, the proliferating cells decreased to 483/944 in H358, 197/926 in H2122, and 570/1,477 in SW1573 (S1 Table). We therefore fitted six separate binary RF classifiers, one for each cell line-time-point dataset, rather than pooling cells across cell lines or treatment times. Model performance using Receiver Operating Characteristic (ROC) curves consistently yielded an Area Under the Curve (AUC) of over 0.85, indicating reliable predictive accuracy. In the SW1573 cell line, the model yielded the AUC of 0.97 at T0 (Fig 1B) and maintained robust performance with the AUC of 0.96 at T72 (Fig 1C). Comparable performance metrics were observed in H358 (S1A Fig) and H2122 cell lines as well (S1B Fig), suggesting that the model reliably captured relevant proliferative signatures across different genetic backgrounds. To further confirm performance, we additionally evaluated precision-recall (PR) curves and out-of-bag (OOB) error rates. The hold-out and OOB estimates were broadly consistent (S2 Table). From 0.791 to 0.998 of hold-out ROC-AUC, and from 0.842 to 0.985 of OOB ROC-AUC values), supporting internal model stability; however, class-specific PR analyses showed weaker performance for the minority cell state in several imbalanced datasets. Thus, retention of genes contributing to the published cell-state annotations may contribute to the high internal discrimination.

**Fig 1:**
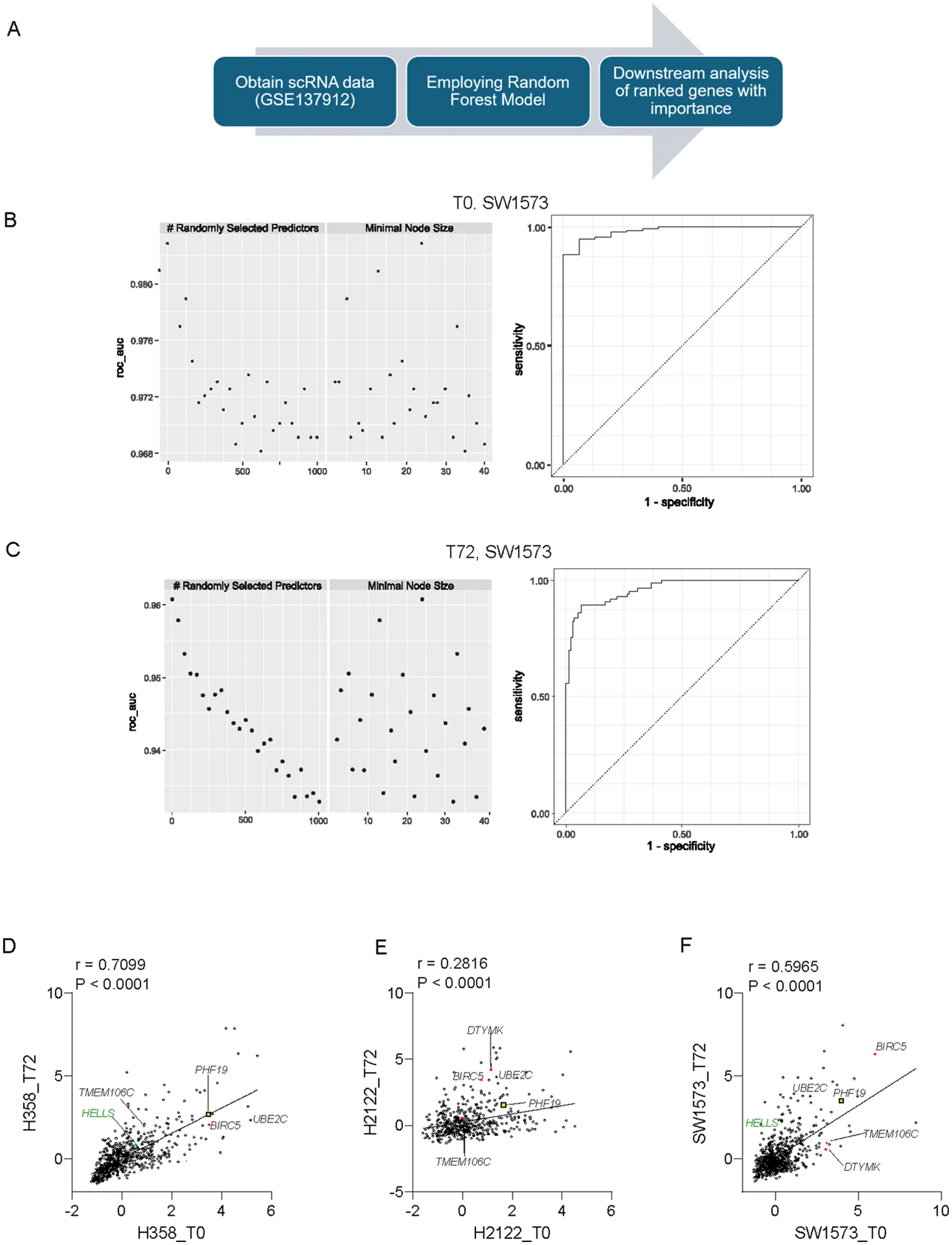
Random Forest workflow and within-dataset model performance. A) Scheme overview of the study workflow. B, C) Trained results of SW1573 treated with ARS-1620 for 0hr (B) and 72hr (C). Left: Hyperparameter optimization plot showing the ROC-AUC score (y-axis) across 1,000 gene features (x-axis) (P < 0.01). Right: The corresponding ROC curve for the optimized model, plotting sensitivity (true positive rate) against 1 – specificity (false positive rate). The dashed diagonal line represents the performance expected by random chance (AUC = 0.50). D – F) Correlations of normalized importance scores for genes shared between T0 and T72 in H358 (D), H2122 (E), and SW1573 (F). Highlighted genes are found in Table 3.

To further assess the model of the gene importance rankings, we examined genes appearing in the top-ranked sets at both T0 and T72 within the same cell line. After fitting each model using the complete set of retained scRNA-seq gene features, we ranked genes according to impurity-based feature importance and selected the top 1,000 genes per cell line and time point for downstream analyses. To evaluate sensitivity to this operational threshold, the downstream analyses were compared between using the top 500 genes and the top 1,000 genes. It produced no material changes in the principal prioritized genes, enriched biological processes, or overall biological interpretation, indicating that the findings were not dependent on the top 1,000 threshold (S3 Table). After we obtained 1000 genes per cell line at T0 and T72, we further selected common genes in both T0 and T72 within the same cell line (659 genes in H358, 478 genes in H2122, 672 genes in SW1573, S1 Data). Next, we computed Pearson correlations of their normalized importance scores across time points (H358: r = 0.71; H2122: r = 0.28; SW1573: r = 0.60; all p < 0.0001, Fig 1D – F), providing an independent layer of validation beyond classification accuracy. This indicates that the importance rankings reflect stable transcriptional features of proliferative cell identity rather than time-point-specific model artifacts. Interestingly, we observed the weaker correlation in H2122 compared to H358 and SW1573, likely implying the more extensive transcriptional reprogramming occurring in H2122. H2122 harbors mutations in *STK11* and *KEAP1* (https://depmap.org/portal/cell_line/ACH-000311?tab=mutations), which are known to alter adaptive responses [28,36,37]. This suggests that the transcriptional state of H2122 at 72 hours more substantially diverges from its own baseline than the other two lines, supported by the findings showing that *STK11*/*KEAP1* co-mutations are associated with reduced response to KRAS inhibitors and altered adaptive dynamics [38,39]. In the performance evaluation, the high AUC values and significant intra-cell-line importance correlations establish that the RF modeling framework is both accurate and reproducible, providing a rigorous foundation for the gene-level biological interpretations presented in the subsequent sections.

### Analysis to identify potential genes contributing to cell state in T0 and T72

Our analysis of the top-ranked genes at T0 revealed a set of genes that is important for cell cycle and division (Fig 2A, S2A Fig, and S1 Data). These genes are predominantly clustered within categories related to cancer cell division: cell cycle, DNA replication, and mitosis (Fig 2B, S2 Data). Next, we compared our top-ranked genes with previously reported genes associated with tumor progression. We observed substantial overlap with the established proliferation drivers, including *H2AFZ*, *CKS1B*, *TUBA1B*, *RRM2*, and *BIRC5* [40]. Our recapitulation with the published genes provided validation of our machine learning approach, which reliably captured bona fide proliferative genes rather than technical artifacts. Furthermore, these consistent findings provided a foundation for identifying early adaptation genes at a later point.

**Fig 2:**
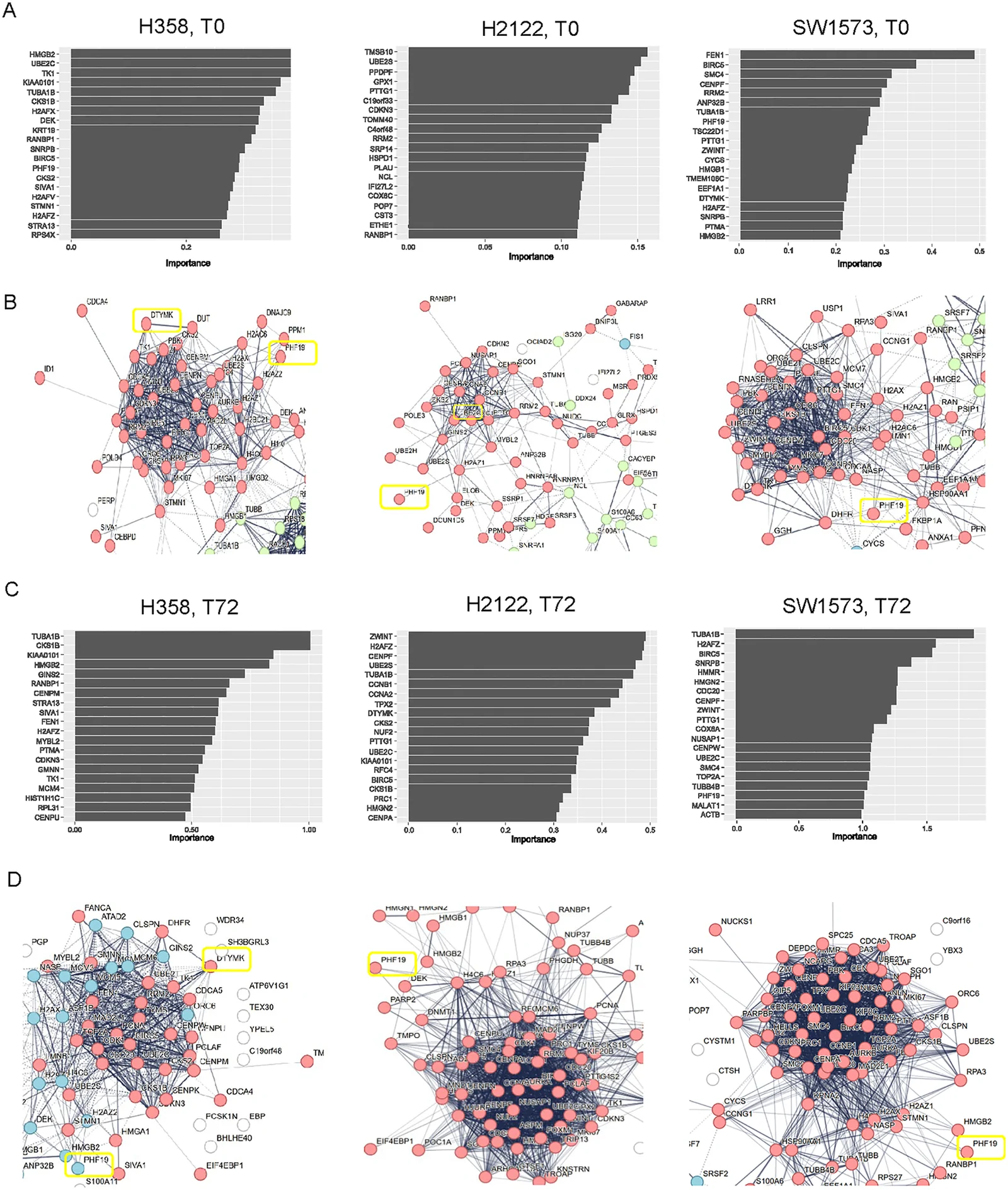
Top 20 genes in three KRASG12C cell lines and their clusters. A, C) Top 20 genes were identified by RF modeling in three cell lines under treatment for 0hr and 72hr (x-axis: the magnitude of importance score). B, D) Clusters in STRING analysis using the top 100 genes from each cell line at T0 (B) and T72 (D). See S5 5A1 – 6 Fig for 100 gene network and their cluster maps. SF5A-1, 2, and 3 correspond to T0 of H358, H2122, and SW1573, respectively in Fig 2B. SF5A-4, 5, and 6 correspond to T72 of H358, H2122, and SW1573, respectively in Fig 2D. The potential emerging contributors are highlighted by a yellow box.

Indeed, our analysis of gene rankings at T72 provided us with informative results. We identified the top 20 most important genes, including *TUBA1B*, *TK1*, *DTYMK*, *HMGB2*, and *H2AFZ* (Table 1), in three independent cell lines (Fig 2C, 2D, S2B Fig, S1 Data, and S2 Data). Notably, while some of these genes were already found at T0 with significance, their persistent or enhanced importance at 72 hours suggests they are critical for sustained proliferation (Fig 2D, S2 Data). Next, as we classified common and unique genes aligned within T72, we identified 397 common genes in three cell lines, prominently enriched in cell cycle and division (S3 Fig, S3 Data). This observation reinforces the idea of a sustained proliferative phenotype under therapeutic pressure. The distinct sets of genes conditionally essential at T72 were *298* in H358, *335* in H2122, and *334* genes in SW1573, distributed across four importance classes (Fig 3A, S3D Fig, and S4 Data). Despite the heterogeneity of the underlying gene sets, functional profiling identified four shared biological processes within upper-middle or top importance classes (Fig 3B, Table 2, S4 Data). These findings suggest that different cell lines use distinct gene sets to achieve shared adaptive responses. Interestingly, these converged processes were prominently enriched in cellular biosynthesis and metabolism pathways (Fig 3B, S4 Data), suggesting that they may be a hallmark of early adaptive responses to counteract therapy-induced stress.

**Table 1:** Top candidate genes at T72 and examples of shared genes in three cell lines.

Rank	H358	H2122	SW1573
1	**TUBA1B**	ZWINT	**TUBA1B**
2	**TK1**	**H2AFZ**	NEAT1
3	DTYMK	UBE2C	**H2AFZ**
4	HMGB2	PRC1	CCNB1
5	**H2AFZ**	**TUBA1B**	HMGB1
6	GINS2	LSM4	AURKB
7	STRA13	MKI67	ZWINT
8	KIAA0101	TPX2	UBE2S
9	TYMS	TUBB	**CKS1B**
10	RANBP1	AURKB	CENPW
11	**CKS1B**	RANBP1	**BIRC5**
12	FEN1	KIAA0101	NUCKS1
13	CENPM	**TK1**	HMGN2
14	PTMA	CENPU	SMC4
15	SNRPB	RPA3	HMMR

**Table 2:** Different genes, shared pathways: Potentially common biological processes and their associated candidate genes in three cell lines under therapeutic pressure at T72.

Associated converged pathways	H358	Tier	H2122	Tier	SW1573	Tier
RNA processing & translation	*RPS7*	Top	*RPL36AL*	Top	*YBX3*	Top
	*RPS23*	Top	*HNRNPA1*	Top	*RPLP1*	Top
	*IARS*	Top	*SRSF5*	Top	*—*	
Redox / detox / oxidative stress	*TXNIP*	Upper-middle	*CYBA*	Top	*EPHX1*	Top
			*GSTP1*	Top	*ATOX1*	Top
Metabolic rewiring	*HINT2*	Top	*ALDOA*	Top	*EPHX1*	Top
	*APOE*	Top	*UGCG*	Top	*—*	
	*ECI1*	Upper-middle	*SDHC*	Top	*—*	
Cytoskeleton / motility	*TAGLN2*	Top	*ARPC3*	Top	*EZR*	Top
	*CORO1B*	Top	*BRK1*	Top	*MYL6B*	Top

* Tier: The genes belong to top or upper-middle class of importance score.

**Fig 3:**
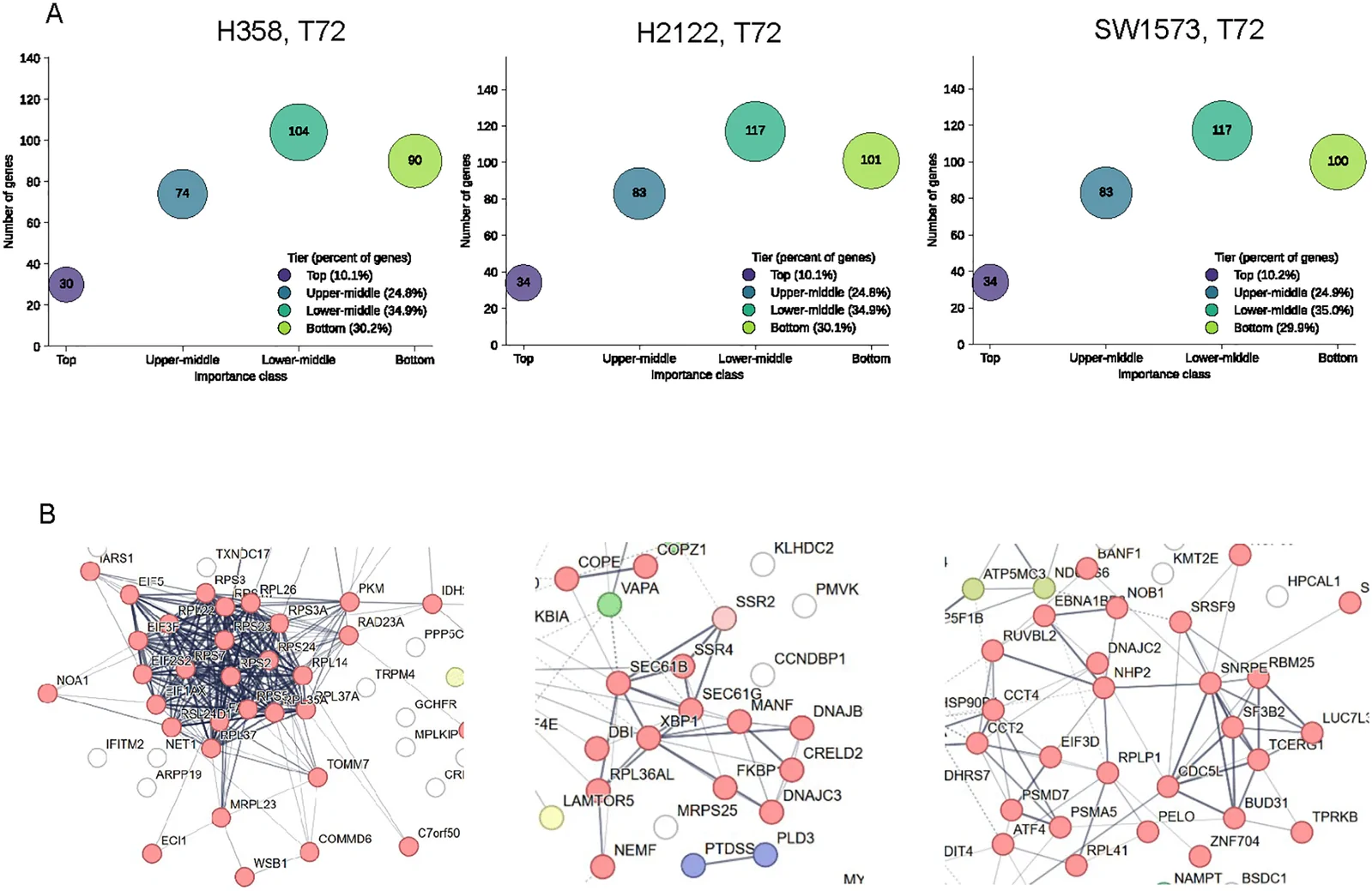
Top-ranked unique genes in the individual cell lines under treatment for 72hr. A) Distribution of unique genes with four importance classes (298 genes in H358, 335 genes in H2122, and 334 genes in SW1573). See S3D Fig for additional support. B) Cluster map of STRING analysis using only top and upper-middle tiers of the unique genes. Multiple interconnected subclusters reflect the multi-pathway nature of the adaptive response (cluster of cytoplasmic translation and ribosome (H358), cluster of protein processing in endoplasmic reticulum and protein export (H2122), and cluster of spliceosomes (SW1573)). See S5B1 – 3 Fig for larger versions of these maps. The list of unique genes and biological processes are shown in S4 Data.

In summary, our analysis of gene rankings captured cells that 1) underwent sustained proliferation, and 2) mounted adaptive responses to therapeutic pressure. These gene sets may collectively drive a temporal shift from the baseline state, representing a non-uniform early adaptation that correlates with the emergence of resistance across three independent cell lines [33,41,42]. Thus, the temporal transition at 72 hours can serve as a critical biological window that distinguishes constitutive growth programs from the active “transcriptional rewiring” required for cells to survive KRAS^G12C^ inhibition.

### Potential molecular regulators overarching early adaptation

Beyond the common biological processes defining the temporal transition, our analysis also highlighted 47 universal genes warranting further investigation (Fig 4A, Table 3). Of 397 at T72 (S3 Fig, S4A Fig, and S3 Data), we selected 139 genes within two upper classes (S4B Fig). By cross examination in three cell lines, 47 genes were true common genes among the top or upper-middle classes, suggesting their role as dominant contributors to cell cycle regulation (Fig 4B). For example, PHF19 (PHD Finger Protein 19), a component of PRC2 complex [43,44], that we identified potentially serves as an emerging molecular regulator in NSCLC. Its normalized importance score was comparable across the cell lines [2.692 (top class), 1.599 (upper-middle class), and 3.532 (top class) in H358, H2122, and SW1573, respectively] (Fig 4C, Table 3). Several reports have suggested its role in different cancers [45,46], but not in NSCLC. Although the normalized importance score of *TMEM106C* (Transmembrane protein 106C, localized in ER) was lower [1.989 (top class), 0.567 (upper-middle class), 0.952 (upper-middle)], in H358, H2122, and SW1573, respectively (Fig 4C, Table 3), TMEM106C also appeared as an emerging regulator [47,48].

**Table 3:** 47 universal genes in three cell lines at T72.

Gene	Norm.Importance_H358	Tier_H358	Norm.Importance_H2122	Tier_H2122	Norm.Importance_SW1573	Tier_SW1573
ATAD2	1.531383	Upper-middle	1.826247	Upper-middle	1.533656	Upper-middle
BIRC5	2.056624	Top	3.452379	Top	6.330339	Top
CACYBP	0.69602	Upper-middle	1.530467	Upper-middle	1.2516	Upper-middle
CDC20	1.472717	Upper-middle	1.800335	Upper-middle	4.865548	Top
CDCA3	0.720126	Upper-middle	1.532957	Upper-middle	2.291748	Top
CDK1	1.675565	Top	1.885902	Upper-middle	2.030049	Upper-middle
CDKN3	3.490097	Top	2.18091	Top	2.953763	Top
CENPA	0.417654	Upper-middle	2.94871	Top	1.204583	Upper-middle
CENPF	0.816855	Upper-middle	5.789251	Top	4.856308	Top
CENPW	2.099336	Top	2.17685	Top	3.797631	Top
CKS1B	7.874937	Top	3.443915	Top	1.13046	Upper-middle
CKS2	1.190025	Upper-middle	4.028413	Top	0.924457	Upper-middle
CLSPN	1.171538	Upper-middle	1.187716	Upper-middle	1.76086	Upper-middle
COX8A	0.597385	Upper-middle	0.552998	Upper-middle	3.933776	Top
DTYMK	2.141761	Top	4.207124	Top	0.581201	Upper-middle
H2AFZ	4.001418	Top	5.846413	Top	6.502264	Top
HELLS	0.852237	Upper-middle	2.398429	Top	1.104521	Upper-middle
HIST1H4C	2.57234	Top	1.330261	Upper-middle	2.343201	Top
HMGB1	2.376184	Top	2.179194	Top	0.878465	Upper-middle
HMGB2	6.210124	Top	2.604996	Top	1.564622	Upper-middle
KIAA0101	6.358764	Top	3.616705	Top	2.023451	Upper-middle
LSM4	1.045166	Upper-middle	1.635796	Upper-middle	1.644935	Upper-middle
MAD2L1	2.494518	Top	2.140066	Upper-middle	1.333652	Upper-middle
NEAT1	1.601022	Upper-middle	0.923534	Upper-middle	1.642943	Upper-middle
NUCKS1	0.626444	Upper-middle	0.735231	Upper-middle	1.718747	Upper-middle
NUSAP1	0.630484	Upper-middle	2.329137	Top	3.863576	Top
PHF19	2.692356	Top	1.599695	Upper-middle	3.53263	Top
PRC1	0.389033	Upper-middle	3.177019	Top	2.363197	Top
PTGES3	0.85758	Upper-middle	1.08178	Upper-middle	1.601713	Upper-middle
PTMA	3.575364	Top	0.566362	Upper-middle	2.326332	Top
RANBP1	4.565436	Top	2.36595	Top	2.004095	Upper-middle
RPL10	2.13576	Top	1.043483	Upper-middle	1.289147	Upper-middle
RRM2	1.417854	Upper-middle	2.679739	Top	1.649775	Upper-middle
S100A6	0.862364	Upper-middle	0.727374	Upper-middle	2.092309	Top
SIVA1	4.117336	Top	1.129252	Upper-middle	0.70128	Upper-middle
SMC4	0.659868	Upper-middle	1.537163	Upper-middle	3.780738	Top
SNRPB	2.685054	Top	2.447482	Top	5.491676	Top
STMN1	1.249923	Upper-middle	2.332197	Top	1.539812	Upper-middle
TMEM106C	1.988816	Top	0.566587	Upper-middle	0.951575	Upper-middle
TOP2A	2.244038	Top	2.766754	Top	3.722348	Top
TUBA1B	7.875921	Top	5.499212	Top	8.069228	Top
TUBB	2.356354	Top	2.817545	Top	1.298218	Upper-middle
TUBB4B	0.446862	Upper-middle	1.215297	Upper-middle	3.671594	Top
UBE2C	2.315577	Top	3.673423	Top	3.786951	Top
UBE2S	2.149312	Top	5.578543	Top	1.108741	Upper-middle
UBE2T	1.309031	Upper-middle	0.969196	Upper-middle	1.437136	Upper-middle
ZWINT	0.702651	Upper-middle	5.895639	Top	4.652842	Top

Norm.importance score was used for the plot Fig 4A (all 47) and 4C (blue selected 6 genes).

* The expression analysis of two emerging regulatory genes (PHF19 and TMEM106C) is present in Table 4.

**Fig 4:**
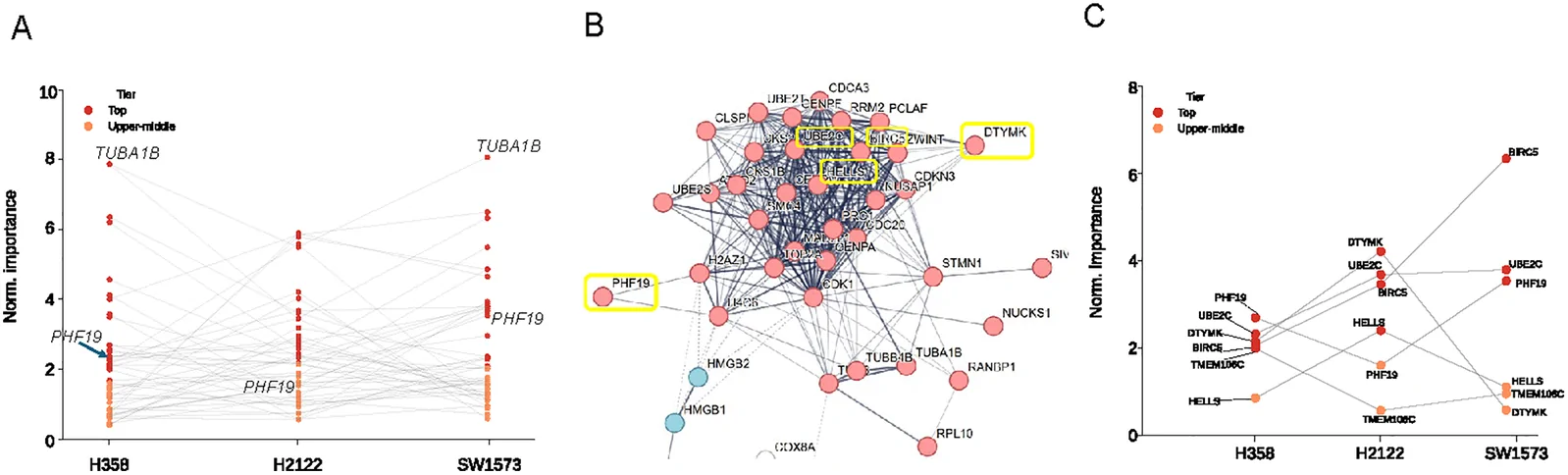
Changes in importance score of 47 shared genes in three cell lines at T72. A) Importance trajectory of 47 genes. **B)** Cluster 1 in STRING analysis using 47 shared genes. See S5C Fig for the full STRING map and clusters. The biological processes are shown in S3C Data) Importance trajectory of selected 6 genes, highlighting emerging and known targets (same as A, but reduced the number of genes to plot).

Cell-state labels (proliferative vs. quiescent) derive from a predefined cell-cycle-phase signature as discussed [33]. These labeling genes were not excluded from the Random Forest’s candidate feature space. We therefore tested what portion of our top 1,000 discriminative gene list overlaps with that predefined signature. 20.0–21.3% of genes across the three cell lines were overlapped whereas the remainder (78.7–80.0%) fell entirely outside the predefined sets (S4 Table). Both PHF19 and TMEM106C fall within this non-overlapping majority (not members of the predefined G0, G1S, S, G2M, M, or MG1 phase-scoring modules). Their prioritization therefore did not result from direct reuse of genes included in the original labeling signatures. We additionally examined the direct expression patterns of PHF19 and TMEM106C independently of their RF importance scores. In all three cell lines at both T0 and T72, both genes showed higher mean normalized expression in cells carrying the published proliferating annotation than in cells annotated as quiescent (Fig 5, Table 4, and S6 Fig). The difference was particularly consistent for PHF19, for which detection frequencies were 27.9–62.4 percentage points higher in proliferating cells across the six models. TMEM106C showed the same directional pattern, although separation was more modest in H2122. These results demonstrate that the RF prioritization of PHF19 and TMEM106C corresponds to directly observable state-associated expression differences. Next, the known targets in NSCLC, including BIRC5 (Baculoviral IAP repeat containing 5, associated with cell cycle/division) [49], UBE2C (Ubiquitin conjugating enzyme E2C, associated with protein homeostasis) [50], DTYMK (Deoxythymidylate kinase, associated with metabolic process) [51], and HELLS (Helicase, lymphoid specific, associated with cell cycle/division) were identified [52], validating the results (Fig 4C, Table 3). Interestingly, *BIRC5* and *UBE2C* maintained the top class of importance across three cell lines, suggesting they act as universal drivers of early adaptation. Additionally, eEF2 (Eukaryotic translation elongation factor 2) emerged as a known target with minor importance regarding translation and transcription regulation. It suggests how eEF2 contextually contributes to facilitating the RNA processing and translation control [53–55] as reprogramming tactic cancer cells use under metabolic or signaling stress to prioritize survival protein synthesis [56–58]. Together, targeting specific molecular candidates such as PHF19, TMEM106C, BIRC5, UBE2C, and eEF2 offers the potential feasibility of developing precision interventions.

**Table 4:** Expression-level analysis of two emerging regulators.

Line	Gene	Mean expression: Quiescent @T0	Mean expression: Proliferative @T0	Mean expression: Quiescent @T72	Mean expression: Proliferative @T72
H358	PHF19	0.458	2.343	0.244	1.13
H2122		0.942	1.521	0.187	0.703
SW1573		0.803	2.325	0.699	2.062
H358	TMEM106C	0.901	2.1	0.756	1.665
H2122		1.887	2.196	1.438	1.882
SW1573		1.628	2.7	1.516	2.243

**Fig 5:**
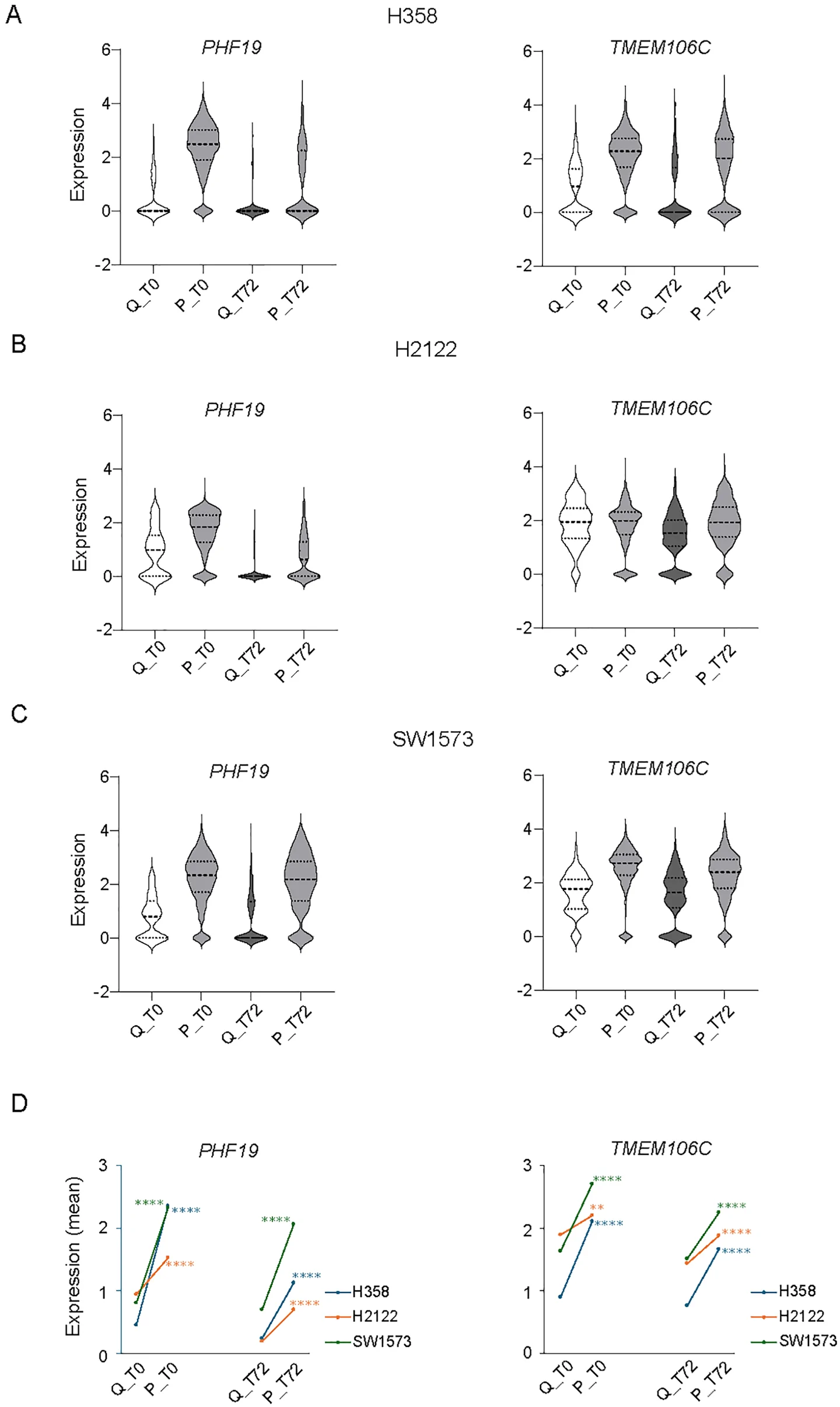
Differential expression of two emerging regulators in three cell lines. A – C) Violin plots showing expression levels of two emerging regulators, *PHF19* and *TMEM106C,* in three cell lines, H358 (A, n = 105 (Q_T0), 974 (P_T0), 461 (Q_T72), and 483 (P_T72)), H2122 (B, n = 70 (Q_T0), 412 (P_T0), 729 (Q_T72), and 197 (P_T72)), and SW1573 (C, n = 88 (Q_T0), 916 (P_T0), 907 (Q_T72), and 570 (P_T72)), (Q_T0 or 72 indicates quiescent cells at T0 or T72. P_T0 or 72 indicates proliferative cells at T0 or T72). D) Trace average expression levels of them in two distinct cell populations at time points. Student t-test, ** *P* < 0.01, **** *P* < 0.0001 (Note: S6 Fig is a comparison of expression level at two different time points (T0 vs. T72) in the same cell populations (quiescent or proliferative)).

## Discussion

Our employment of the Random Forest model and its importance rankings revealed a correlation in established mechanisms of cancer cell proliferation (T0) and early adaptive responses (T72) in KRAS^G12C^ lung cancer cell lines. The identification of unique gene signatures at T72 provides important insights into adaptive response mechanisms. The temporal shift from baseline (T0) to T72 can serve as a biological window that distinguishes constitutive growth programs from the active transcriptional rewiring required for cells to survive KRAS^G12C^ inhibition. While baseline data identified universal proliferation drivers, the T72 marks captured cells that had successfully mounted adaptive responses to therapeutic pressure. The converged pathways underlying the unique gene sets (Table 2) appear to mediate the early adaptation that may compensate for KRAS inhibition, rather than simply maintaining baseline proliferative programs [59]. Thus, intervening in the shared biological processes may provide an opportunity to manipulate early adaptive responses, possibly leading to interception of resistance before it matures [60,61]. While targeting broad processes provides a valid therapeutic framework, the clinical feasibility of such an approach can be difficult to assess without defined effectors.

To explore alternative strategies to intervene in the converged pathways, targeting molecular candidates within these pathways offers a more feasible and precise means of intervention as we identified interesting molecular targets such as PHF19, TMEM106C, BIRC5, UBE2C, and eEF2. The consistent prioritization of these candidates across divergent genetic backgrounds indicates a conserved, high-priority axis that governs the transition toward early adaptation. Two predefined proliferative BIRC5 and UBE2C emerged as universal contributors in top importance class across all three cell lines, suggesting a core adaptive mechanism centered on apoptosis evasion and cell cycle maintenance. While regulators like eEF2 facilitate the biosynthetic shifts required for survival, the consistent prioritization of BIRC5 (an IAP family member) and UBE2C (a mitotic regulator) implies that maintaining cell viability and genomic stability is a prerequisite for the maturation of drug resistance. Mechanistically, resistance development may be achieved through therapeutic escape by enhancing their protein synthesis capacity, potentially allowing them to more efficiently produce bypass pathway components or anti-apoptotic proteins [62–64]. Simultaneously, PHF19 and TMEM106C also emerged as universal contributors within the top or upper-middle importance class across all three cell lines. While BIRC5 and UBE2C maintain cell viability and mitotic stability as they belong to the predefined gene sets (proliferative), PHF19 and TMEM106C fall outside of predefined quiescent or proliferative gene sets. PHF19 may provide the epigenetic framework necessary to sustain these adaptive gene programs and TMEM106C may facilitate tumor motility to evade hostile environments such as KRAS inhibition, suggesting their contribution to a multi-layered ‘survival module’ required for early adaptation. Taken together, these early adaptive responses and their associated molecular contributors represent high-priority targets for experimental validation in the future as a potential strategy to intercept resistance prior to its onset [60,61,65]. Since data from the dependency map (https://depmap.org) classified *PHF19* as neither essential nor selective, it will serve as a suitable molecular target. On the other hand, *BIRC5* is categorized as a common essential gene, and *UBE2C* and *TMEM106C* as strongly selective genes, suggesting that mitigating off-target cytotoxicity will be a key challenge for their therapeutic application. *eEF2* is also a common essential gene, however, targeting eEF2K (eEF2 regulatory kinase) showed preclinical effectiveness in different cancers [66,67], providing a proof-of-concept approach to mitigate off-target cytotoxicity. Successfully targeting them will provide the possibility of rational combination strategies by pairing KRAS^G12C^ inhibitors, supported by the study showing eEF2K responds to KRAS^G12D^ inhibition [68].

However, we acknowledge important limitations of this study, warranting further investigation. First, we employed only one model using the top 1,000 genes after fitting, possibly leading to bias, compared to the seven different models used in the previous study [40]. Although the RF model we used successfully recapitulated the previous findings, we cannot exclude the possibility of inherent bias. The performance analyses showed that the RF model tells the published proliferating and quiescent annotations within each condition-specific dataset. The broad concordance between hold-out and OOB estimates provides counter-insights into performance being driven by one favorable test split although the available data do not contain independent biological or sequencing replicates. Cells in the training, validation, test, and OOB evaluations share the same condition-specific experimental and technical context. These metrics therefore quantify internal within-dataset discrimination and should not be interpreted as cross-batch or independent biological generalizability. Another limitation is the relationship between the outcomes and the inputs. The cell-state annotations were derived from predefined cell-cycle modules, and genes from these modules were retained in the input genes, 8,687. Recovery of these genes is biologically coherent and provides an internal consistency check, however, it can also contribute to high classification performance. Accordingly, neither ROC-AUC nor overlap with known proliferation genes establishes that the importance rankings identify causal regulators. Impurity importance is unsigned and can also distribute importance among correlated genes. Therefore, ranked genes should be interpreted as features associated with the classified state. Third, our analysis relies on only two time points (0 hours and 72 hours), which may not fully capture the dynamic temporal evolution of early adaptation. The 72-hour time point may represent an early adaptive response rather than established resistance, and critical transcriptional changes may occur at intermediate time points or beyond 72 hours, which are not captured in this study. The key difference between our study and the previous study [40] is that we focused on early adaptation rather than resistance development. Our shift in focus is supported by two main observations: 1) treatment window, 72 hr is not generally sufficient to develop resistance, and 2) ARS-1620, the compound used in the original scRNA-seq (GSE137912), is unlikely to maintain high concentration over 72 hr, further supporting that the identified changes precede resistance development or maturation. ARS-1620 shows approximately 60% oral bioavailability in mice and hour-long effective half-life [10], suggesting the majority of free compound would be cleared or sequestered over multi-day exposure. Without experimental evidence, it is unclear whether the important genes we and others found may ultimately contribute to resistance development or early adaptation. Nevertheless, they conceptually align more closely with early adaptation responses. Furthermore, future studies incorporating additional time points or using acquired resistance models – cell lines that have developed stable resistance over prolonged KRAS^G12C^ inhibitor exposure – would provide a more comprehensive view of the early adaptation and resistance development. Fourth, our findings are based on *in vitro* cell line models even without control cell lines, which may not fully recapitulate the complexity of resistance development observed in a clinical setting. The tumor microenvironment, immune interactions, and tumor heterogeneity present in clinical samples are absent from the 2D cell line system. While our computational approach identified candidate early adaptive genes, functional validation through genetic or pharmacological perturbation is required to establish causal roles in preventing or delaying resistance development by interfering with the early adaptation. Finally, but not least, our study examined only three cell lines, all harboring the same KRAS^G12C^ mutation. Resistance mechanisms may differ across other KRAS variants or in tumors with different co-occurring mutations. Thus, using 3D culture (e.g., organoids with different KRAS backgrounds) and at least two different KRAS^G12C^ (or ^G12D^) inhibitors may partly overcome such limitations in future.

## Conclusion

This study represents an important step forward in understanding the molecular basis of KRAS^G12C^ inhibitor resistance in non-small cell lung cancer. By applying machine learning to RNA sequencing data across two time points and three cell line models, we identified both known essential proliferation genes and emerging candidate adaptive genes. Our findings suggest that cancer cells initiate adaptive programs to evade KRAS^G12C^ inhibition through rewiring compensatory pathways. The models showed stable internal discrimination by hold-out and OOB evaluation, while class-specific precision-recall analysis highlighted limitations arising from cell-state imbalance. Ultimately, this work will contribute to the broader goal of developing more effective, durable interventions for KRAS-mutant NSCLC by intercepting resistance before it matures. As KRAS^G12C^ inhibitors become standard of care for lung cancer patients, understanding and targeting resistance development will be essential for maximizing clinical benefit and improving outcomes in this challenging disease. Therefore, independent datasets and functional perturbation will be required to determine whether these candidates contribute causally to treatment adaptation or represent actionable vulnerabilities.

## Supporting information

S1 Fig Random Forest model performance. A, B) Trained results of H358 (A) and H2122 (B) treated with ARS-1620 for 0hr and 72hr. Left: Hyperparameter optimization plot showing the ROC-AUC score (y-axis) across 1,000 gene features (x-axis), with models achieving statistical significance at P < 0.01. Right: The corresponding ROC curve for the optimized model, plotting sensitivity (true positive rate) against 1 – specificity (false positive rate). The curve demonstrates strong model performance, with an AUC of over 0.85 in H358 (A), and an AUC of over 0.8 in H2122 (B) in both T0 and T72. The dashed diagonal line represents the performance expected by random chance (AUC = 0.50).
(PDF)

S2 Fig Random Forest model performance. A, B) Tested results of ROC curves show sensitivity vs. 1-specificity in three cell lines (H358, H2122, and SW1573) under treatment for 0hr (A), and 72hr (B). The consistently high AUC values across all three cell lines confirm that the model is not overfit to the training data and its ability to discriminate between proliferative and quiescent cells.
(PDF)

S3 Fig Top-ranked 397 common genes in all three cell lines at T72. A) Venn diagram to summarize the number of genes, 298 in H358 only, 335 in H2122 only, 334 in SW1573 only, 152 in H358 & H2122, 153 in H358 & SW1573, 116 in H2122 & SW1573, and 397 in three lines. B) Distribution of 397 shared genes with the four classes, Top (n = 40, 10.1%), Upper-Middle (n = 99, 24.9%), Lower-Middle (n = 139, 35.0%), and Bottom (n = 119, 30.0%). The 40 top-class and 99 upper-middle-class genes, which together comprise the most functionally impactful portion of the common set, provide the basis for the downstream analyses to identify universal adaptive targets (see Fig 4). C) STRING analysis using the common genes. 397 common genes encode not only proliferation machinery but also biosynthetic and proteostatic maintenance under therapeutic pressure. The biological processes are shown in S3D Data) Stacked bar plot of distribution of the number of genes.
(ZIP)

S4 Fig Changes in importance score of common genes in three cell lines at T72. A, B) Importance trajectory of the shared genes. Parallel coordinate plots display the normalized RF importance scores of all shared 397 genes (A) in top (red), upper-middle (orange), lower-middle (yellow), and bottom classes (blue) and shared 139 genes from 397 that achieve top or upper-middle class (B).
(PDF)

S5 Fig Enlarged STRING maps and clusters. **A1 – 3**) STRING maps and their clusters of top 100 genes in T0 of H358. H2122, and SW1574. **A4 – 6**) STRING maps and their clusters of top 100 genes in T72 of H358. H2122, and SW1574. **B1 – 3**) STRING maps and their clusters of unique top and upper-middle class genes in T72 of H358. H2122, and SW1574. **C**) STRING maps and their clusters of shared 47 genes.
(ZIP)

S6 Fig Trace average expression levels of PHF19 and TMEM106C at two distinct time points in the cell populations. Student t-test, ns *P* > 0.05, ** *P* < 0.01, **** *P* < 0.0001 (Note: Fig 5 D is a comparison of expression level in two different cell populations (quiescent vs. proliferative) at the same time point (T0 or T72)).
(PDF)

S1 Table Number of genes in cell states. (XLSX)

S2 Table Extra layer validation in RF modeling. (XLSX)

S3 Table Sensitivity test in the top 500 and the top 1,000 genes. (XLSX)

S4 Table Number of gene indicating cell states in the top 1,000 genes. (XLSX)

S1 Data Gene list in three cell lines at T0 and T72. (XLSX)

S2 Data Biological processes in three cell lines at T0 and T72. (XLSX)

S3 Data Common genes across three cell lines at T72 and their biological processes. (XLSX)

S4 Data Unique genes in individual cell lines at T72 and their biological processes. (XLSX)
