# Supplementary material for: Random forest approach to predict genes correlated with adaptive responses to KRASG12C inhibitor
Source: PLoS One. 2026 Nov 11;21(11):e0359938. doi: 10.1371/journal.pone.0359938 (PMC13754168; doi:10.1371/journal.pone.0359938)

Supplementary Figure 3

D

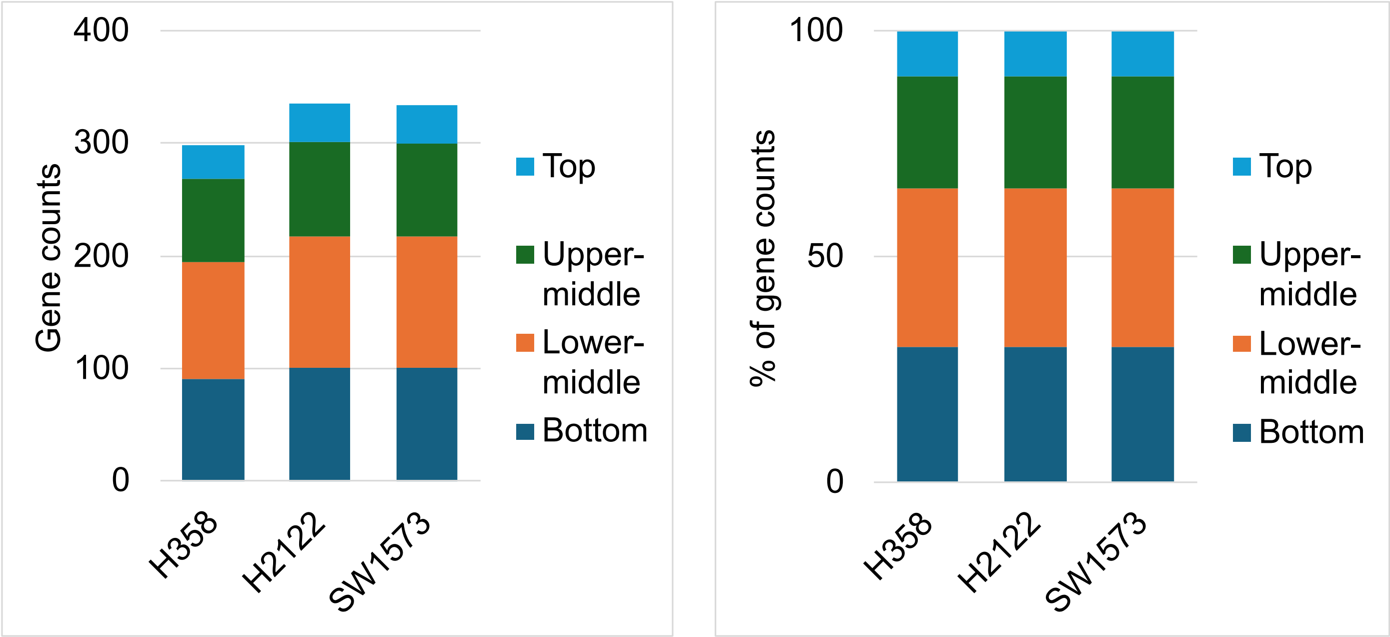

Supplement: Supplementary file 5 [file pone.0359938.s003.zip › SF3D.Re.pdf]
