# Supplementary material for: Random forest approach to predict genes correlated with adaptive responses to KRASG12C inhibitor
Source: PLoS One. 2026 Nov 11;21(11):e0359938. doi: 10.1371/journal.pone.0359938 (PMC13754168; doi:10.1371/journal.pone.0359938)
Supplement: Supplementary file 6 [file pone.0359938.s005.zip › SF5A-3.Re.pdf]

Supplementary Figure 5

A3

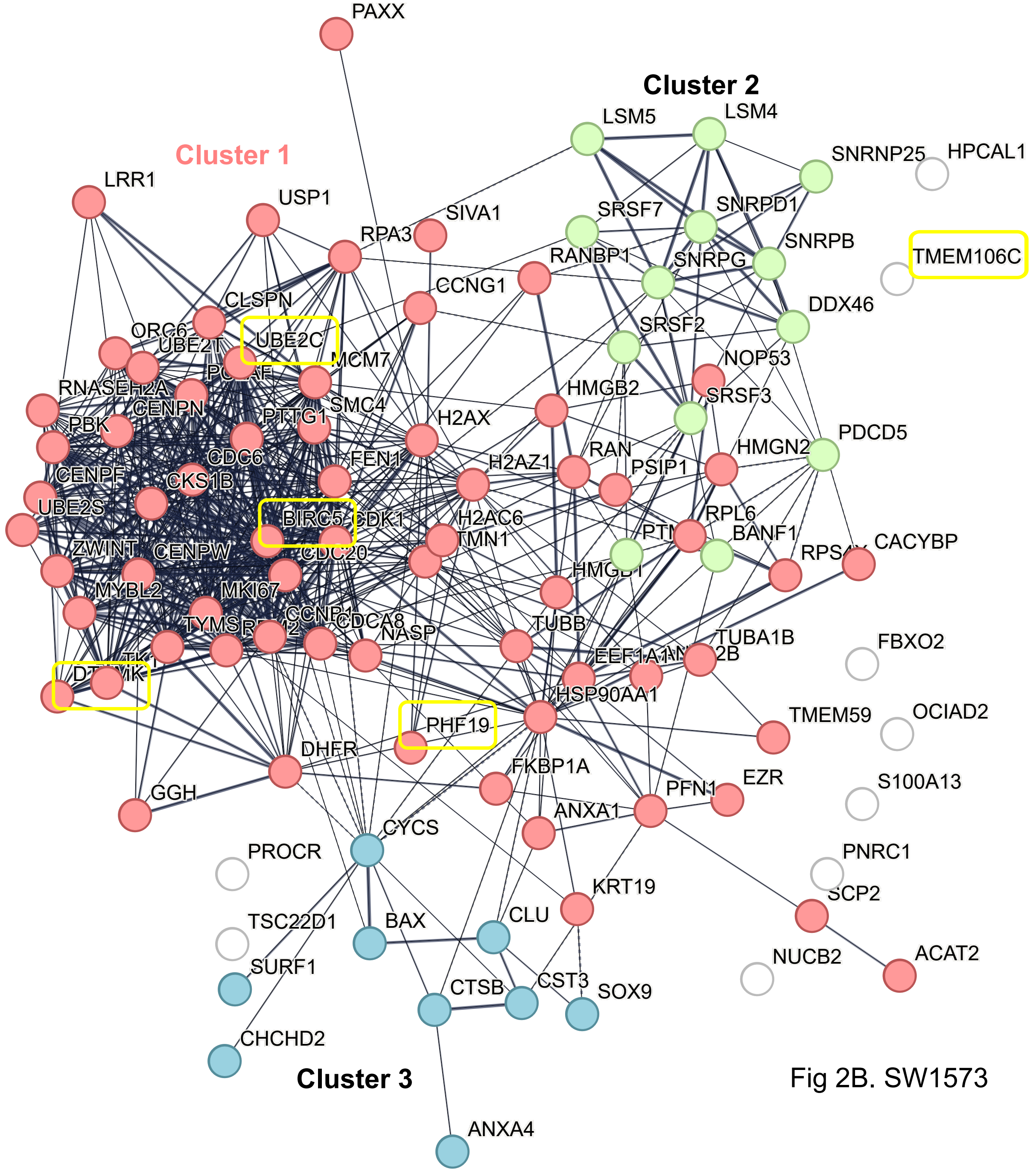

Fig 2B. SW1573
