# Supplementary material for: Random forest approach to predict genes correlated with adaptive responses to KRASG12C inhibitor
Source: PLoS One. 2026 Nov 11;21(11):e0359938. doi: 10.1371/journal.pone.0359938 (PMC13754168; doi:10.1371/journal.pone.0359938)
Supplement: Peer review history

# Peer review: Decision Letter 0

**DOI:** 10.1371/journal.pone.0359938.r001

**Authors:** Bing He

20 May 2026

Dear Dr. Lee,

Thank you for submitting your manuscript to PLOS ONE. After careful consideration, we feel that it has merit but does not fully meet PLOS ONE’s publication criteria as it currently stands. Therefore, we invite you to submit a revised version of the manuscript that addresses the points raised during the review process.

Please submit your revised manuscript by Jul 04 2026 11:59PM. If you will need more time than this to complete your revisions, please reply to this message or contact the journal office at plosone@plos.org. When you're ready to submit your revision, log on to https://www.editorialmanager.com/pone/ and select the 'Submissions Needing Revision' folder to locate your manuscript file.

- A letter that responds to each point raised by the academic editor and reviewer(s). You should upload this letter as a separate file labeled 'Response to Reviewers'.
- A marked-up copy of your manuscript that highlights changes made to the original version. You should upload this as a separate file labeled 'Revised Manuscript with Track Changes'.
- An unmarked version of your revised paper without tracked changes. You should upload this as a separate file labeled 'Manuscript'.

If you would like to make changes to your financial disclosure, please include your updated statement in your cover letter. Guidelines for resubmitting your figure files are available below the reviewer comments at the end of this letter.

If applicable, we recommend that you deposit your laboratory protocols in protocols.io to enhance the reproducibility of your results. Protocols.io assigns your protocol its own identifier (DOI) so that it can be cited independently in the future. For instructions see: https://journals.plos.org/plosone/s/submission-guidelines#loc-laboratory-protocols. Additionally, PLOS ONE offers an option for publishing peer-reviewed Lab Protocol articles, which describe protocols hosted on protocols.io. Read more information on sharing protocols at https://plos.org/protocols?utm_medium=editorial-email&utm_source=authorletters&utm_campaign=protocols.

As the corresponding author, your ORCID iD is verified in the submission system and will appear in the published article. PLOS supports the use of ORCID, and we encourage all coauthors to register for an ORCID iD and use it as well. Please encourage your coauthors to verify their ORCID iD within the submission system before final acceptance, as unverified ORCID iDs will not appear in the published article. *Only*  the individual author can complete the verification step; PLOS staff *cannot*  verify ORCID iDs on behalf of authors.

We look forward to receiving your revised manuscript.

Kind regards,

Bing He

Academic Editor

PLOS One

Journal Requirements:

When submitting your revision, we need you to address these additional requirements.

1. Please ensure that your manuscript meets PLOS ONE's style requirements, including those for file naming. The PLOS ONE style templates can be found at

https://journals.plos.org/plosone/s/file?id=wjVg/PLOSOne_formatting_sample_main_body.pdf and

https://journals.plos.org/plosone/s/file?id=ba62/PLOSOne_formatting_sample_title_authors_affiliations.pdf.

2. Please note that PLOS One has specific guidelines on code sharing for submissions in which author-generated code underpins the findings in the manuscript. In these cases, we expect all author-generated code to be made available without restrictions upon publication of the work. Please review our guidelines at https://journals.plos.org/plosone/s/materials-and-software-sharing#loc-sharing-code and ensure that your code is shared in a way that follows best practice and facilitates reproducibility and reuse.

3. Thank you for stating the following financial disclosure:

“P30CA016059: Massey Comprehensive Cancer Center - Cancer Center Support Grant “

Please state what role the funders took in the study.  If the funders had no role, please state: "The funders had no role in study design, data collection and analysis, decision to publish, or preparation of the manuscript."

If this statement is not correct you must amend it as needed.

Please include this amended Role of Funder statement in your cover letter; we will change the online submission form on your behalf.

4. If the reviewer comments include a recommendation to cite specific previously published works, please review and evaluate these publications to determine whether they are relevant and should be cited. There is no requirement to cite these works unless the editor has indicated otherwise.

[Note: HTML markup is below. Please do not edit.]

Reviewers' comments:

Reviewer's Responses to Questions

**Comments to the Author**

1. Is the manuscript technically sound, and do the data support the conclusions?

Reviewer #1: Yes

Reviewer #2: Yes

**********

2. Has the statistical analysis been performed appropriately and rigorously?

Reviewer #1: I Don't Know

Reviewer #2: Yes

**********

3. Have the authors made all data underlying the findings in their manuscript fully available?

The PLOS Data policy

Reviewer #1: Yes

Reviewer #2: Yes

**********

4. Is the manuscript presented in an intelligible fashion and written in standard English?

Reviewer #1: Yes

Reviewer #2: Yes

**********

Reviewer #1: 1. The authors employed the random forest machine learning algorithm to classify cells into proliferative and quiescent states. The detailed classification results of proliferative and quiescent cell states based on the GSE137912 dataset should be presented, and the specific implementation procedures of the classification method need to be supplemented in detail.

2. Was the gene list input into the random forest model derived from the entire transcriptome or only differentially expressed genes? Was 1000 genes ultimately used for the analysis? If so, such a gene number is unconventional, and the rationality behind this selection should be explicitly explained. If subsequent results are established based on this 1000-gene list, sufficient evidence must be provided to demonstrate that such a large gene set will not lead to overinterpretation of the results.

3. Did the authors infer cell proliferative and quiescent states using predefined gene sets? The study also identified an additional gene set that can distinguish these two cell states. What is the unique advantage of this newly identified gene list? The scientific innovation and significance of this additional gene signature should be fully elaborated in the manuscript. Although several individual genes have been mentioned by referring to previous literature, it is still suggested to analyze the expression patterns of newly identified representative candidate genes and related pathways in the original GSE137912 dataset. Moreover, validation using external datasets and experimental verification are both indispensable to support the findings.

4. A large number of network interaction diagrams were displayed in the main text, but these diagrams lack sufficient interpretability and discriminative power. It is recommended to replace the original network plots with subnetwork analysis or functional enrichment analysis results in the main figures.

Reviewer #2: 1. General Summary

In this manuscript, the authors present a machine learning framework using Random Forest (RF) to analyze single-cell RNA sequencing (scRNA-seq) data across three KRASG12C mutant non-small cell lung cancer (NSCLC) cell lines (H358, H2122, and SW1573) treated with the KRAS inhibitor ARS-1620. The study aims to delineate the transcriptomic landscape during the early adaptive window (72 hours post-treatment) prior to the establishment of overt, mature drug resistance. By employing the built-in feature importance capabilities of RF, the authors systematically rank and categorize genes essential for classifying proliferative versus quiescent cell states into specific tier levels.

The methodology provides a solid computational proof of concept for identifying key transcriptional drivers, successfully recapitulating well-documented cancer essential genes and proliferation markers (such as H2AFZ, CKS1B, TUBA1B, RRM2, and BIRC5) at baseline. Crucially, the model uncovers a shared survival strategy across diverse genetic backgrounds, highlighting novel overarching early adaptive genes such as PHF19 and TMEM106C. This study offers valuable, actionable insights for future translational research, providing a powerful conceptual framework for intercepting drug resistance through proactive, rational combination therapies targeting early adaptive programs rather than fully established, refractory resistance states. The topic is highly relevant to precision oncology, and the computational workflow is well-integrated; however, several major methodological concerns and data visualization clarity issues must be addressed before publication.

2. Major Comments & Critical Revisions

1. Overfitting and High-Performance Evaluation: The model reports exceptionally high classification metrics, with Area Under the Curve (AUC) values reaching 0.96 to 0.97 for the SW1573 cell line and similarly high performance across other lines. Given these near-perfect values, the authors must explicitly address potential data leakage or model overfitting. Please provide details on how cell-to-cell dependencies within the same scRNA-seq batch were handled during data splitting, and present additional evaluation metrics (e.g., precision-recall curves or out-of-bag error rates) to rigorously confirm model generalizability.

2. Absence of Raw Expression Traceability: The authors successfully utilize the Random Forest algorithm as an elegant feature selector/indicator machine to pinpoint biologically important genes. However, there is a distinct lack of raw data abundance or baseline transcriptomic visualization to trace back these selected features. To substantiate that these top-ranked genes represent biological reality rather than artifactual modeling partitioning, the authors should provide at least one main text figure (such as a volcano plot, differential expression heatmap, or split violin plots) showcasing the direct abundance/expression differences of these high-importance features between the two key operational time points T0 and T72

3. Verbose Figure Legends vs. Methodology Text: The figure legends are overly verbose and contain detailed descriptions of statistical parameters and analytical tools that belong in the main text. Technical descriptions of the analytical parameters, normalization procedures, and pipeline packages should be migrated out of the captions and consolidated within the Materials and Methods section to streamline readability.

3. Minor Comments & Technical Clarifications

1. Data Splitting Randomization: In the Methods section, the authors state that the dataset was split into a 70-15-15% ratio for training, validation, and testing subsets. Please clarify the exact randomization strategy utilized. Was stratified sampling applied based on cell lines, sequencing depth, or cell-state labels to ensure balanced splits?

2. Hyperparameter and Training Details: To ensure scientific reproducibility, please supplement the training description with explicit hyperparameter values. Specifically, state the number of trees (ntree), the number of variables randomly sampled at each split (mtry), the minimum node size, and the explicit convergence criteria utilized within the ranger engine framework.

3. Figure 1 Revisions: Figure 1A: The current workflow flowchart is text-heavy and visually overwhelming. Please simplify the descriptive text boxes into an intuitive, schematic layout, utilizing standard biological/computational icons to cleanly convey the underlying computational pipeline steps.

o Figures 1B & 1C: To maximize readability, clearly add prominent titles or headers for T0 and T72 directly onto the respective ROC subplots.

4. Figure 2 Revision (Venn Diagram): The presentation of unique and overlapping gene identification across the cell lines would be significantly enhanced by visual data structures. Please implement a classical Venn diagram (or an UpSet plot) to explicitly illustrate the intersection and cell-line-specific counts of the selected important genes across the three distinct experimental cell lines.

5. Figure 3 Revision (Bubble Plot Alternative): The current bubble plot carries a highly limited density of informational value and fails to clearly delineate the variance across classification tiers. It is strongly recommended to replace or supplement this with a structured bar chart complete with standard error bars to properly show the expression scales and distribution across the 4 categorical importance tiers across all 3 cell lines.

6. Figures 2 & 3 (STRING Network Visualizations): The text labels within the STRING network interaction diagrams are far too small, rendering them illegible. Please adjust the graphic resolution or node label size so that readers can identify the critical hub genes.

o Please explicitly highlight or visually isolate the key adaptive genes discussed in the text within these networks.

o A clear color-coded legend defining the edge/node meanings must be added directly to the figure panel, rather than relying solely on network plots without annotations.

7. Table 3 Redundancy: Table 3 contains highly redundant columns repeating identical gene names or synonymous annotations. Please clean up the matrix structure to merge or remove duplicate identifier columns to ensure a concise presentation of the key targets.

**********

what does this mean?). If published, this will include your full peer review and any attached files.

If you choose “no”, your identity will remain anonymous but your review may still be made public.

**Do you want your identity to be public for this peer review?**  For information about this choice, including consent withdrawal, please see our Privacy Policy

Reviewer #1: No

Reviewer #2: **Yes:**  Ziyang Gao

**********

[NOTE: If reviewer comments were submitted as an attachment file, they will be attached to this email and accessible via the submission site. Please log into your account, locate the manuscript record, and check for the action link "View Attachments". If this link does not appear, there are no attachment files.]

To ensure your figures meet our technical requirements, please review our figure guidelines: https://journals.plos.org/plosone/s/figures

You may also use PLOS’s free figure tool, NAAS, to help you prepare publication quality figures: https://journals.plos.org/plosone/s/figures#loc-tools-for-figure-preparation.

NAAS will assess whether your figures meet our technical requirements by comparing each figure against our figure specifications.

# Author comment: Author response to Decision Letter 1

**DOI:** 10.1371/journal.pone.0359938.r002

18 Aug 2026

FYI. A letter of response to reviewers is attached.

Response to reviewers

PONE-D-26-14468

Random Forest approach to predict genes correlated with adaptive responses to KRASG12C inhibitor

PLOS One

Summary

We thank the Academic Editor and both reviewers for their careful and constructive evaluation. The manuscript has been revised extensively to clarify the published cell-state annotations, model construction, data splitting, hyperparameters, performance evaluation, feature-ranking strategy, direct expression support, network presentation, figures, tables, and study limitations. The detailed point-by-point responses are provided below.

These major revisions include:

• Clarification that proliferating and quiescent labels were obtained directly from the published GSE137912 cell annotations, with complete cell counts reported for all six datasets (three cell lines x two time points).

• Explicit documentation of the 70:15:15 stratified split, fixed random seed, ranger hyperparameters, bootstrap settings, and OOB evaluation.

• Addition of class-specific precision-recall curves, PR-AUC values, OOB ROC-AUC, OOB Brier scores, and OOB misclassification errors.

• Clarification that all 8,687 retained gene-expression features entered each model and that the top 1,000 genes were selected only after model fitting as an inclusive interpretive threshold.

• Direct expression-level visualization of PHF19 and TMEM106C across cell lines, time points, and published cell states.

• Replacement of dense main-text STRING maps with readable k-means-defined clusters, while retaining complete networks in the Supporting Information.

• Addition of a supplementary Venn diagram, replacement of the tier bubble plot with stacked bars, simplification of Figure 1, shortening of figure legends, and restructuring of Table 3.

• Removal or qualification of statements implying ground truth, independent validation, causal function, or established therapeutic targets.

Responses to Reviewer 1

1. The authors employed the random forest machine learning algorithm to classify cells into proliferative and quiescent states. The detailed classification results of proliferative and quiescent cell states based on the GSE137912 dataset should be presented, and the specific implementation procedures of the classification method need to be supplemented in detail.

Response: We thank the reviewer for raising the point. We understand there is a methodological gap. We have substantially expanded the Methods (cell state labeling) and Results. Cell-state labels were obtained directly from the cell-level annotations accompanying GSE137912 (Ref # 33. Xue et al. PMID: 31915379). We did not recalculate cell-cycle scores or reassign states. Cells annotated as G0 were treated as quiescent, whereas cells annotated as G1S, S, G2M, M, or MG1 were treated as proliferating. The final dataset contained 5,912 cells: H358 T0, 974 proliferating and 105 quiescent; H358 T72, 483 and 461; H2122 T0, 412 and 70; H2122 T72, 197 and 729; SW1573 T0, 916 and 88; and SW1573 T72, 570 and 907, respectively. We fitted six separate binary classifiers, one for each cell line-time-point combination, using the 8,687 retained expression features as predictors and the published binary Mode annotation as the outcome.

2. Was the gene list input into the random forest model derived from the entire transcriptome or only differentially expressed genes? Was 1000 genes ultimately used for the analysis? If so, such a gene number is unconventional, and the rationality behind this selection should be explicitly explained. If subsequent results are established based on this 1000-gene list, sufficient evidence must be provided to demonstrate that such a large gene set will not lead to overinterpretation of the results.

Response: We thank the reviewer for requesting clarification regarding the gene features used in the Random Forest analysis and the rationale for retaining 1,000 genes. We have revised the Materials and Methods and Results sections to make this distinction explicit. We clarified that the RF input was not restricted to differentially expressed genes. All 8,687 processed gene-expression features retained in GSE137912 were entered into each classifier. The top 1,000 genes were selected only after model fitting from a single unsigned impurity-based importance ranking for each model. Thus, the top 1,000 cutoff was selected as an exploratory threshold sufficiently broad to preserve coordinated pathway-level signals distributed across correlated genes, rather than the number of genes initially supplied to the classifier. To assess whether the biological conclusions were dependent on this threshold, we repeated the downstream analyses using the top 500 importance-ranked genes. The top 500 and top 1,000 analyses produced no material differences in the principal genes, enriched biological processes, or overall interpretation. We have added this sensitivity analysis to the revised manuscript. We have also clarified that the top 1,000 genes do not represent 1,000 statistically significant or causally validated genes. Rather, they constitute an exploratory importance-ranked candidate set. Biological interpretation was focused on reproducibly prioritized genes, cross-cell-line overlap, importance tiers, and consistently enriched pathways, while the functional roles of individual candidates require independent experimental validation.

3. Did the authors infer cell proliferative and quiescent states using predefined gene sets? The study also identified an additional gene set that can distinguish these two cell states. What is the unique advantage of this newly identified gene list? The scientific innovation and significance of this additional gene signature should be fully elaborated in the manuscript. Although several individual genes have been mentioned by referring to previous literature, it is still suggested to analyze the expression patterns of newly identified representative candidate genes and related pathways in the original GSE137912 dataset. Moreover, validation using external datasets and experimental verification are both indispensable to support the findings.

Response: We thank the reviewer for this question, which led us to directly quantify cell classification not previously reported. Cell-state labels derive from a predefined cell-cycle-phase signature as discussed in the first comment (R1-1), and these labeling genes were not excluded from the Random Forest's candidate feature space. We therefore checked what fraction of our top1,000 discriminative gene list overlaps with that predefined signature: 20.0–21.3% across the three cell lines, with the remainder (78.7–80.0%) falling entirely outside the predefined sets. Both PHF19 and TMEM106C, the two candidates central to our claim, fall within this non-overlapping majority (not members of the predefined G0, G1S, S, G2M, M, or MG1 phase-scoring modules). Their prioritization therefore did not result from direct reuse of genes included in the original labeling signatures. We now report this overlap analysis explicitly in the Result section (Table below), framing the partial overlap as an internal validation signal (recovery of known biology) alongside the larger, independent discovery, and have added expression-level analysis of these two genes within GSE137912 to further substantiate the finding independent of RF importance scores. We have also valued our Limitations to state plainly that external-dataset and experimental validation remain necessary future steps.

We also added direct expression-level analyses of PHF19 and TMEM106C within GSE137912, comparing their normalized expression and detection frequencies between the published proliferating and quiescent cell populations. This analysis provides expression-level support that is separate from the RF feature-importance ranking, although it remains an analysis of the same underlying dataset. (new main figure 5 and new table 4)

We identified no independent dataset that reproduced the complete experimental design of GSE137912, including the same cell lines, inhibitor exposure, 72-hour time point, single-cell resolution, and published proliferating/quiescent annotations. One independent GSE183516 is available to see H358 ARS-1620 RNA-seq dataset. However, because this dataset is bulk RNA-seq and contains earlier treatment times (up to 24hr), the analysis limits us to validate the single-cell classifier. We also agree that functional perturbation will be required to determine causality, but new experimental studies are beyond the scope of this current computational reanalysis. Accordingly, we have removed causal and therapeutic language and now present the genes as candidates requiring future functional validation (novel therapeutic targets that control adaptive resistance � “PHF19 and TMEM106C are candidate regulators whose manipulation may alter adaptive cell-state behavior.”)

4. A large number of network interaction diagrams were displayed in the main text, but these diagrams lack sufficient interpretability and discriminative power. It is recommended to replace the original network plots with subnetwork analysis or functional enrichment analysis results in the main figures.

Response: We agree that full STRING maps in the main text added visual density without proportional interpretive value, and that the enrichment statistics, which actually convey significance and biological specificity , were previously confined to supplementary tables disconnected from the figures. We have replaced the primary network figures in Figures 2B & D, 3B, and 4B with representative clusters. Full STRING maps remain available in Supplement Figure 5 (SF5A1 – 6, SF5B1 – 3, and SF5C) for readers who want the complete interaction data.

Responses to Reviewer 2, Dr. Ziyang Gao

Reviewer #2: 1. General Summary

In this manuscript, the authors present a machine learning framework using Random Forest (RF) to analyze single-cell RNA sequencing (scRNA-seq) data across three KRASG12C mutant non-small cell lung cancer (NSCLC) cell lines (H358, H2122, and SW1573) treated with the KRAS inhibitor ARS-1620. The study aims to delineate the transcriptomic landscape during the early adaptive window (72 hours post-treatment) prior to the establishment of overt, mature drug resistance. By employing the built-in feature importance capabilities of RF, the authors systematically rank and categorize genes essential for classifying proliferative versus quiescent cell states into specific tier levels. The methodology provides a solid computational proof of concept for identifying key transcriptional drivers, successfully recapitulating well-documented cancer essential genes and proliferation markers (such as H2AFZ, CKS1B, TUBA1B, RRM2, and BIRC5) at baseline. Crucially, the model uncovers a shared survival strategy across diverse genetic backgrounds, highlighting novel overarching early adaptive genes such as PHF19 and TMEM106C. This study offers valuable, actionable insights for future translational research, providing a powerful conceptual framework for intercepting drug resistance through proactive, rational combination therapies targeting early adaptive programs rather than fully established, refractory resistance states. The topic is highly relevant to precision oncology, and the computational workflow is well-integrated; however, several major methodological concerns and data visualization clarity issues must be addressed before publication.

We thank Reviewer 2 for the positive assessment of the biological question and computational framework and for the detailed recommendations that improved methodological transparency and figure readability.

Major 1. Overfitting and High-Performance Evaluation: The model reports exceptionally high classification metrics, with Area Under the Curve (AUC) values reaching 0.96 to 0.97 for the SW1573 cell line and similarly high performance across other lines. Given these near-perfect values, the authors must explicitly address potential data leakage or model overfitting. Please provide details on how cell-to-cell dependencies within the same scRNA-seq batch were handled during data splitting, and present additional evaluation metrics (e.g., precision-recall curves or out-of-bag error rates) to rigorously confirm model generalizability.

Response: We agree with the reviewer's concern that near-perfect AUC values warrant scrutiny. Because GSE137912 contains no independent replicates, batch-level splitting was not applicable. Cells were assigned to mutually exclusive training, validation, and test sets, without cell shared across partitions and no test-set use during tuning. However, all cells were in distinct conditions, which were two time-points T0, and T72, three different cell lines, and two cell classifications (proliferative vs. quiescent) in the scRNA-seq dataset. We therefore interpret the AUC values as within-dataset discrimination, not evidence of independent biological or cross-batch generalizability. Now we have revised the manuscript accordingly.

For more details, each of the six-cell line-time-point datasets was partitioned independently into mutually exclusive training, validation, and test subsets in the revised analysis. We therefore interpret the reported metrics as within-dataset performance rather than evidence of independent biological or cross-batch generalizability. We added class-specific precision-recall curves and PR-AUC values for proliferating and quiescent cells, together with OOB ROC-AUC, PR-AUC, Brier score, and misclassification error. Held-out ROC-AUC values ranged from 0.791 to 0.998, and OOB ROC-AUC values ranged from 0.842 to 0.985. The held-out and OOB estimates were broadly concordant, supporting internal model stability; however, class-specific PR analyses showed weaker performance for the minority cell state in several imbalanced datasets. We removed statements that high ROC-AUC establishes biological ground truth or independent validation and now acknowledge that retention of genes contributing to the published cell-state annotations may contribute to the high internal discrimination. We also acknowledge that genes contributing to the published cell-state annotations were retained among the predictors, which may contribute to the high internal discrimination and further limits causal interpretation of model performance.

Major 2. Absence of Raw Expression Traceability: The authors successfully utilize the Random Forest algorithm as an elegant feature selector/indicator machine to pinpoint biologically important genes. However, there is a distinct lack of raw data abundance or baseline transcriptomic visualization to trace back these selected features. To substantiate that these top-ranked genes represent biological reality rather than artifactual modeling partitioning, the authors should provide at least one main text figure (such as a volcano plot, differential expression heatmap, or split violin plots) showcasing the direct abundance/expression differences of these high-importance features between the two key operational time points T0 and T72

Response: We agree that direct expression-level visualization was needed to connect the RF importance rankings to the underlying transcriptomic data. We therefore added a new main figure (Figure 5), a new Table 4, and a new Supplementary figure 6 showing PHF19 and TMEM106C expression at T0 and T72 in H358, H2122, and SW1573, stratified by the published proliferating and quiescent annotations, which Reviewer 1 asked too (R1-3: direct expression-level analyses of PHF19 and TMEM106C within GSE137912).

This analysis demonstrates that both candidates show directly observable state- and time-associated expression patterns independent of the RF importance scores. Because the dataset lacks independent biological replicates, we present these results descriptively and do not use cell-level significance testing as evidence of biological replication.

Major 3. Verbose Figure Legends vs. Methodology Text: The figure legends are overly verbose and contain detailed descriptions of statistical parameters and analytical tools that belong in the main text. Technical descriptions of the analytical parameters, normalization procedures, and pipeline packages should be migrated out of the captions and consolidated within the Materials and Methods section to streamline readability.

Response: We agree with the point. We shortened all main and supplementary figure legends to retain only panel descriptions, visual encodings, abbreviations, and essential sample information. Details of normalization, software, model parameters, data splitting, performance evaluation, statistical procedures, STRING settings, and clustering were moved to the Materials and Methods. Interpretive statements were moved to the Results or Discussion, and unsupported statements that the figures confirm absence of overfitting were removed.

Minor 1. Data Splitting Randomization: In the Methods section, the authors state that the dataset was split into a 70-15-15% ratio for training, validation, and testing subsets. Please clarify the exact randomization strategy utilized. Was stratified sampling applied based on cell lines, sequencing depth, or cell-state labels to ensure balanced splits?

Response: We clarified the randomization strategy in the revised Methods. Each of the six-cell line–time-point datasets was partitioned independently into mutually exclusive training, validation, and test subsets at an approximately 70:15:15 ratio using a fixed random seed of 12345. Sampling was stratified by the published binary cell-state label to preserve the proliferating-to-quiescent ratio across subsets. Cell line and time point were not used as stratification variables because a separate model was constructed for each cell line–time-point combination. Sequencing depth was not used for stratification. We also confirmed that no cell identifier occurred in more than one subset.

Minor 2. Hyperparameter and Training Details: To ensure scientific reproducibility, please supplement the training description with explicit hyperparameter values. Specifically, state the number of trees (ntree), the number of variables randomly sampled at each split (mtry), the minimum node size, and the explicit convergence criteria utilized within the ranger engine framework.

Response: We expanded the Methods to report the complete Random Forest specification. Probability forests were fitted with the ranger engine using 1,000 trees, mtry = 93, and a minimum node size of 1. The mtry value corresponds to the integer square root of the 8,687 retained expression features. Trees used the Gini impurity split rule, unrestricted depth, and bootstrap sampling with replacement at a sampling fraction of 1.0. No class or case weights were applied. Impurity-based variable importance was calculated, probability estimation was enabled, and a fixed forest seed of 12345 was used. Random Forest construction does not use an iterative numerical convergence threshold; training terminated after the prespecified 1,000 trees had been grown. OOB predictions were generated automatically from cells omitted from the bootstrap sample for each tree (please see the response to Major 1).

Minor 3. Figure 1 Revisions:

• Figure 1A: The current workflow flowchart is text-heavy and visually overwhelming. Please simplify the descriptive text boxes into an intuitive, schematic layout, utilizing standard biological/computational icons to cleanly convey the underlying computational pipeline steps.

• Figures 1B & 1C: To maximize readability, clearly add prominent titles or headers for T0 and T72 directly onto the respective ROC subplots.

Response: Figure 1A has been simplified to show only essential steps. Figures 1B/1C and the corresponding Supplement Figure panels now carry on-plot T0/T72 titles, so each ROC curve is identifiable without consulting the caption.

Minor 4. Figure 2 Revision (Venn Diagram): The presentation of unique and overlapping gene identification across the cell lines would be significantly enhanced by visual data structures. Please implement a classical Venn diagram (or an UpSet plot) to explicitly illustrate the intersection and cell-line-specific counts of the selected important genes across the three distinct experimental cell lines.

Response: We agree that a Venn diagram plot improves clarity. We added a three-set Venn diagram to the Supporting Information showing the overlap among the T72 top 1,000 RF-ranked gene lists for H358, H2122, and SW1573. The diagram displays 298 H358-specific genes, 335 H2122-specific genes, 334 SW1573-specific genes, pairwise-only intersections of 152, 153, and 116 genes, and 397 genes shared by all three cell lines. The main text clarifies that 47 of the 397 shared genes additionally met the top or upper-middle importance criterion in all three cell lines. The Venn diagram was placed in the Supporting Information (Supplement figure 3) to avoid overcrowding the main figure.

Minor 5. Figure 3 Revision (Bubble Plot Alternative): The current bubble plot carries a highly limited density of informational value and fails to clearly delineate the variance across classification tiers. It is strongly recommended to replace or supplement this with a structured bar chart complete with standard error bars to properly show the expression scales and distribution across the 4 categorical importance tiers across all 3 cell lines.

Response: Thank you for raising clarity concerns in main Figure 3A. To provide better delineation, now we present stacked bar plots as a new supplementary figure (SF3D). The stacked bar plots show the number of genes in each tier and their %. The near-identical percentage breakdown across cell lines follows directly from our fixed-percentile tier definition (applied independently per cell line) rather than reflecting an independent biological finding, and that error bars are not included because each count is a single-model point estimate rather than a distribution across resamples.

Minor 6. Figures 2 & 3 (STRING Network Visualizations): The text labels within the STRING network interaction diagrams are far too small, rendering them illegible. Please adjust the graphic resolution or node label size so that readers can identify the critical hub genes.

• Please explicitly highlight or visually isolate the key adaptive genes discussed in the text within these networks.

• A clear color-coded legend defining the edge/node meanings must be added directly to the figure panel, rather than relying solely on network plots without annotations.

Response: Thank you for identifying the limited readability. We revised Figures 2 and 3 to display only the selected functionally coherent subnetworks, with enlarged node labels and improved resolution. The key adaptive genes discussed in the text are now explicitly highlighted. The complete STRING networks, including all identified clusters, are retained as high-resolution supplementary figures with distinct clusters. The criteria used to select the subnetworks shown in the main figures are now described in the Materials and Methods and figure legends.

Minor 7. Table 3 Redundancy: Table 3 contains highly redundant columns repeating identical gene names or synonymous annotations. Please clean up the matrix structure to merge or remove duplicate identifier columns to ensure a concise presentation of the key targets.

Response: Thank you for pointing out the redundancy. We reformatted Table 3 accordingly. Now we removed two additional columns of “gene” to remain it as a single column. We retained all other columns, for example, the per-cell-line tier columns, as tier assignment (Top vs. Upper-Middle) is not uniform across the three cell lines for a subset of genes.

Attachment Submitted filename: Response to reviewers_PONE-D-26-14468.docx

# Peer review: Decision Letter 1

**DOI:** 10.1371/journal.pone.0359938.r003

**Authors:** Bing He

21 Sep 2026

Random Forest approach to predict genes correlated with adaptive responses to KRASG12C inhibitor

PONE-D-26-14468R1

Dear Dr. Lee,

We’re pleased to inform you that your manuscript has been judged scientifically suitable for publication and will be formally accepted for publication once it meets all outstanding technical requirements.

Within one week, you’ll receive an e-mail detailing the required amendments. When these have been addressed, you’ll receive a formal acceptance letter and your manuscript will be scheduled for publication.

An invoice will be generated when your article is formally accepted. Please note, if your institution has a publishing partnership with PLOS and your article meets the relevant criteria, all or part of your publication costs will be covered. Please make sure your user information is up-to-date by logging into Editorial Manager at Editorial Manager® and clicking the ‘Update My Information' link at the top of the page. For questions related to billing, please contact billing support.

If your institution or institutions have a press office, please notify them about your upcoming paper to help maximize its impact. If they’ll be preparing press materials, please inform our press team as soon as possible -- no later than 48 hours after receiving the formal acceptance. Your manuscript will remain under strict press embargo until 2 pm Eastern Time on the date of publication. For more information, please contact onepress@plos.org.

Kind regards,

Bing He

Academic Editor

PLOS One

Additional Editor Comments (optional):

Reviewers' comments:

Reviewer's Responses to Questions

**Comments to the Author**

Reviewer #1: All comments have been addressed

Reviewer #2: All comments have been addressed

**********

2. Is the manuscript technically sound, and do the data support the conclusions?

Reviewer #1: Yes

Reviewer #2: Yes

**********

3. Has the statistical analysis been performed appropriately and rigorously?

Reviewer #1: Yes

Reviewer #2: Yes

**********

4. Have the authors made all data underlying the findings in their manuscript fully available?

The PLOS Data policy

Reviewer #1: Yes

Reviewer #2: Yes

**********

5. Is the manuscript presented in an intelligible fashion and written in standard English?

Reviewer #1: Yes

Reviewer #2: Yes

**********

Reviewer #1: (No Response)

Reviewer #2: (No Response)

**********

what does this mean?). If published, this will include your full peer review and any attached files.

If you choose “no”, your identity will remain anonymous but your review may still be made public.

**Do you want your identity to be public for this peer review?**  For information about this choice, including consent withdrawal, please see our Privacy Policy

Reviewer #1: No

Reviewer #2: **Yes:**  Ziyang Gao

**********

# Editor report: Acceptance letter

**DOI:** 10.1371/journal.pone.0359938.r004

**Authors:** Bing He

PONE-D-26-14468R1

PLOS One

Dear Dr. Lee,

I'm pleased to inform you that your manuscript has been deemed suitable for publication in PLOS One. Congratulations! Your manuscript is now being handed over to our production team.

At this stage, our production department will prepare your paper for publication. This includes ensuring the following:

* All references, tables, and figures are properly cited

* All relevant supporting information is included in the manuscript submission,

* There are no issues that prevent the paper from being properly typeset

You will receive further instructions from the production team, including instructions on how to review your proof when it is ready. Please keep in mind that we are working through a large volume of accepted articles, so please give us a few days to review your paper and let you know the next and final steps.

Lastly, if your institution or institutions have a press office, please let them know about your upcoming paper now to help maximize its impact. If they'll be preparing press materials, please inform our press team within the next 48 hours. Your manuscript will remain under strict press embargo until 2 pm Eastern Time on the date of publication. For more information, please contact onepress@plos.org.

You will receive an invoice from PLOS for your publication fee after your manuscript has reached the completed accept phase. If you receive an email requesting payment before acceptance or for any other service, this may be a phishing scheme. Learn how to identify phishing emails and protect your accounts at https://explore.plos.org/phishing.

If we can help with anything else, please email us at customercare@plos.org.

Thank you for submitting your work to PLOS One and supporting open access.

Kind regards,

PLOS One Editorial Office Staff

on behalf of

Dr. Bing He

Academic Editor

PLOS One
